# Discoidin Domain Receptors: Potential Actors and Targets in Cancer

**DOI:** 10.3389/fphar.2016.00055

**Published:** 2016-03-14

**Authors:** Hassan Rammal, Charles Saby, Kevin Magnien, Laurence Van-Gulick, Roselyne Garnotel, Emilie Buache, Hassan El Btaouri, Pierre Jeannesson, Hamid Morjani

**Affiliations:** Extracellular Matrix and Cellular Dynamics, Faculty of Pharmacy, MEDyC Centre National de la Recherche Scientifique UMR7369Reims, France

**Keywords:** discoidin domain receptors, tyrosine kinase, extracellular matrix, collagen, cell signaling, cancer, targeted therapy

## Abstract

The extracellular matrix critically controls cancer cell behavior by inducing several signaling pathways through cell membrane receptors. Besides conferring structural properties to tissues around the tumor, the extracellular matrix is able to regulate cell proliferation, survival, migration, and invasion. Among these receptors, the integrins family constitutes a major class of receptors that mediate cell interactions with extracellular matrix components. Twenty years ago, a new class of extracellular matrix receptors has been discovered. These tyrosine kinase receptors are the two discoidin domain receptors DDR1 and DDR2. DDR1 was first identified in the *Dictyostelium discoideum* and was shown to mediate cell aggregation. DDR2 shares highly conserved sequences with DDR1. Both receptors are activated upon binding to collagen, one of the most abundant proteins in extracellular matrix. While DDR2 can only be activated by fibrillar collagen, particularly types I and III, DDR1 is mostly activated by type I and IV collagens. In contrast with classical growth factor tyrosine kinase receptors which display a rapid and transient activation, DDR1 and DDR2 are unique in that they exhibit delayed and sustained receptor phosphorylation upon binding to collagen. Recent studies have reported differential expression and mutations of DDR1 and DDR2 in several cancer types and indicate clearly that these receptors have to be taken into account as new players in the different aspects of tumor progression, from non-malignant to highly malignant and invasive stages. This review will discuss the current knowledge on the role of DDR1 and DDR2 in malignant transformation, cell proliferation, epithelial to mesenchymal transition, migratory, and invasive processes, and finally the modulation of the response to chemotherapy. These new insights suggest that DDR1 and DDR2 are new potential targets in cancer therapy.

## Introduction

While advances in treatment have increased the survival rate for many cancers, it is still one of the leading causes of death in the world, particularly in developing countries. Cancer represents a tremendous burden on patients, families and societies. Yet, it is generally accepted that cancer risk actually depends on a combination of genes aspects, environment and lifestyle. After surgery, radiation therapy (RT) has long been an integral component of cancer care. It is usually employed to locally eradicate tumor cells as well as alter tumor stroma with either curative or palliative intent. Despite many improvements (image guided radiotherapy, intensity modulated radiotherapy, etc.), RT often fails to provide local tumor control, and delivering higher doses of radiation alone is unlikely to solve this problem (Higgins et al., 2015). In addition, neither surgery nor radiotherapy could control the metastatic spread of tumor. Therefore, current efforts have been focusing on understanding the molecular, cellular, and systemic processes driving cancer initiation, progression, heterogeneity, and metastatic spread.

The extracellular matrix (ECM) critically controls cancer cell behavior by inducing several signaling pathways. Besides conferring structural properties to tissues, ECM is able to regulate cell proliferation, survival, migration, and invasion (Lu et al., 2012). As a major part of the tumor ECM, type I collagen exhibits high density and altered architecture in malignant cancer and is causally linked to tumor formation and metastasis (Provenzano et al., 2006, 2008). Until recently, these effects on tumor cells were exclusively attributed to integrins; a major class of receptors that mediate cell interactions with extracellular matrix components. The identification of the Discoidin Domain Receptor (DDR) family as collagen receptors represents a new paradigm in the regulation of collagen-cell interactions and regulation of tumor progression.

DDR1 and DDR2 were initially discovered by homology cloning based on their catalytic kinase domains and were orphan receptors until 1997 when Shrivastava and co-workers, and Vogel and co-workers, reported that different types of collagen are functional ligands for these receptors (Shrivastava et al., 1997; Vogel et al., 1997). Indeed, DDRs belong to the large family of receptor tyrosine kinases based on the presence of a catalytic kinase domain with a distinct extracellular Discoidin (DS) homology domain (Johnson et al., 1993; Alves et al., 1995; Perez et al., 1996). DDR1 was first identified in the *Dictyostelium discoideum* and was shown to mediate cell aggregation (Breuer and Siu, 1981; Springer et al., 1984). DDR2 shares highly conserved sequences with DDR1 (Carafoli et al., 2009). Both receptors are activated upon binding to collagen. DDR1 is activated by various types of collagen including type I, IV, V, VI, and VIII, whereas DDR2 is only activated by fibrillar collagens, in particular collagens type I, III, and type X (Shrivastava et al., 1997; Vogel et al., 1997; Leitinger and Kwan, 2006). In contrast with classical growth factor tyrosine kinase receptors such as the epithelial growth factor receptor (EGFR) and fibroblast growth factor receptor (FGFR) which display a rapid and transient activation (Dengjel et al., 2007), DDR1 and DDR2 are unique in that they exhibit remarkably delayed and sustained receptor phosphorylation upon binding to collagen (Vogel et al., 1997). Furthermore, many classical tyrosine kinase receptors (RTKs) undergo negative regulation such as receptor/ligand internalization and subsequent degradation or dephosphorylation by phosphatases (Avraham and Yarden, 2011). In the case of DDRs, phosphorylation levels may persist up to 18 h (Vogel et al., 1997).

Both DDRs are expressed early during embryonic development as demonstrated in many *in vivo* studies (Valiathan et al., 2012). Indeed mice lacking DDR1 or DDR2 exhibit major defects in skeletal development (Bargal et al., 2009), reproduction (Matsumura et al., 2009; Kano et al., 2010), inflammation (Olaso et al., 2011), and cardiovascular system (Franco et al., 2010). In addition, they are uniquely positioned to function as sensors for ECM and to regulate a wide range of cell functions such as migration, cell proliferation, cytokine secretion, and ECM homeostasis/remodeling (Valiathan et al., 2012). While activation of DDRs is required for normal development, studies have reported differential expression and mutations of DDR1 and DDR2 in several cancers (Valiathan et al., 2012). In malignant transformation, cell proliferation, epithelial to mesenchymal transition, migration, and invasive processes, the role of DDRs in different aspects of tumor progression will be highlighted. We further discuss recent studies on DDRs as a therapeutic and potential target in cancer but also its role in the modulation of the response to chemotherapy. Hopefully, these useful updates will encourage more research on DDRs in cancer and the possibility to better identify them as promising targets for future therapies.

## Structure, function and regulation of DDRs (Figure 1)

Structurally, DDRs are characterized by 4 different domains: an extracellular region composed of an N-terminal DS domain and a DS-like domain which binds to collagen. DDR1 and DDR2 share high degree of sequence identity in DS and DS-like domains with 59 and 51% of similarity, respectively (Carafoli and Hohenester, 2013). The juxtamembrane (JM) domain is composed of extracellular JM regions of about 50 amino acids for DDR1 and 30 for DDR2 followed by large cytosolic JM regions of about 171 amino acids for DDR1, depending on the protein isoform, and 142 for DDR2 (Leitinger, 2011; Carafoli and Hohenester, 2013). Finally, the catalytic tyrosine kinase domain (KD) is composed of 300 amino acids, undergoes phosphorylation and activates downstream signaling. This KD is ended by a short C-terminal peptide of about 8 amino acids for DDR1 and 6 amino acids for DDR2 (Carafoli and Hohenester, 2013). For detailed structural studies, readers are invited to refer to the cited references (Carafoli et al., 2009, 2012; Fu et al., 2013; Li et al., 2015).

**Figure 1:**
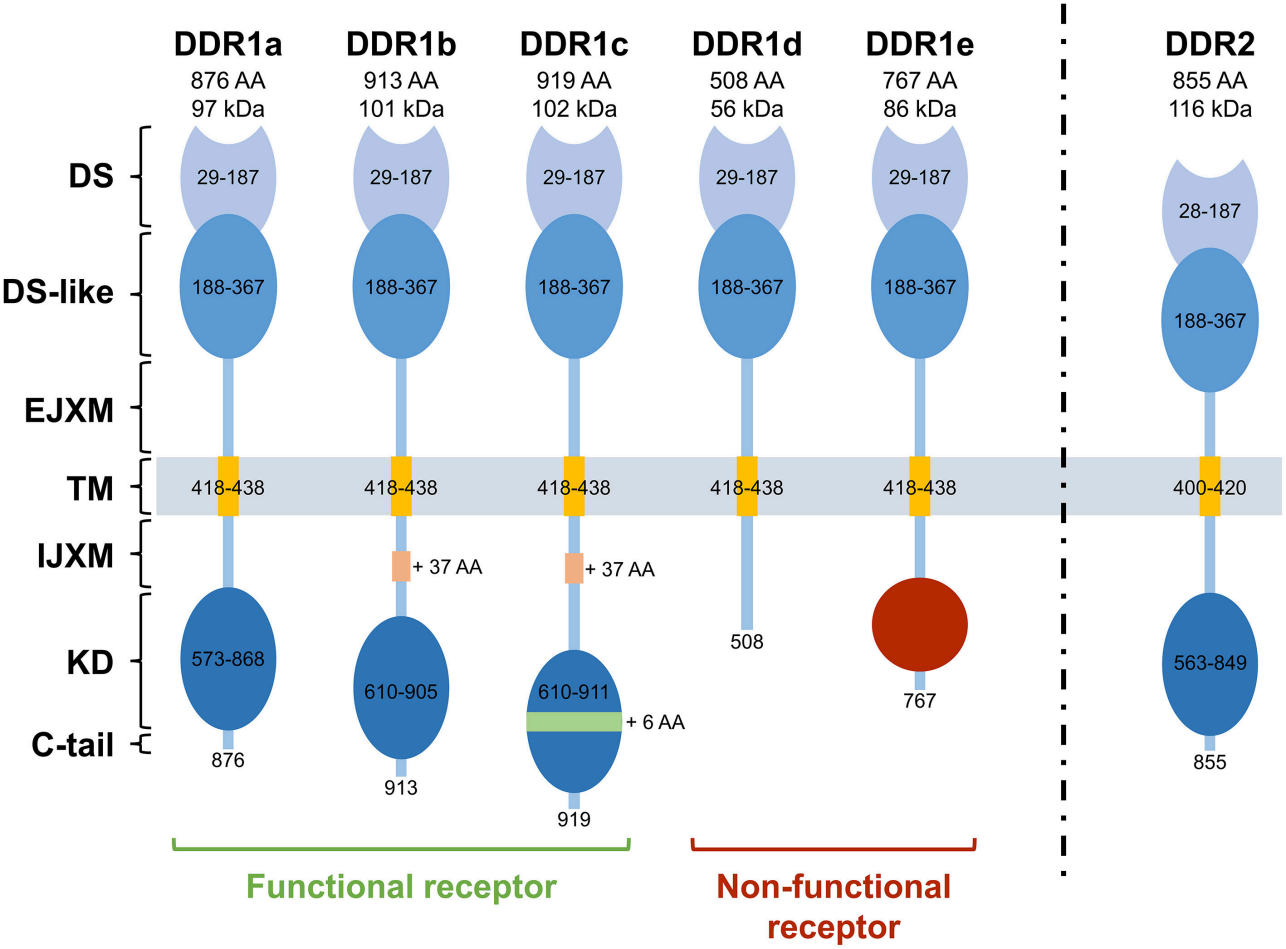
**Structure of the different Discoidin Domain Receptors**. DDR1a, DDR1b, DDR1c, and DDR2 are enzymatic active receptors, and DDR1d and DDR1e are inactive kinase-deficient receptors. DS, discoidin domain; DS-like, discoidin-like domain; EJXM, extracellular juxtamembrane region; TM, transmembrane segment; IJXM, intracellular juxtamembrane region; KD, kinase domain; AA, Amino Acid.

Unlike DDR2, five isoforms of DDR1 (DDR1a, b, c, d, and e) have been described as they exhibit differences in the extent of glycosylation (Vogel, 1999), phosphorylation (Vogel et al., 2006; Carafoli and Hohenester, 2013), protein interactions (Matsuyama et al., 2003), expression patterns, and functions (Vogel et al., 2006). DDR1a, b, and c are kinase-active, whereas DDR1d and e are kinase-deficient because of frame shift and truncation (Alves et al., 2001). While the longest isoform (DDR1c) is composed of 919 amino acids, DDR1a and DDR1b, the most abundant isoforms, lack 37 amino acids in the JM domain or 6 amino acids in the KD. DDR1d and DDR1e isoforms are C-terminally truncated receptors. DDR1d lacks exons 11 and 12 causing a frame-shift mutation that generates a stop codon and premature termination of transcription, whereas DDR1e lacks exons 11 and 12 as well as the first half of exon 10 (Alves et al., 1995, 2001). In 2006, a sixth isoform that lacks a part of the extracellular domain has been described in the postmeiotic germ cells of the rat testis (Mullenbach et al., 2006).

DDRs control important aspects of cell behavior including proliferation, migration, adhesion, and ECM remodeling but are dysregulated in various human diseases. They are both activated by several types of collagen. However, this activation strictly requires collagen to be in its native and triple-helical conformation. Heat-denatured collagen is not recognized by DDRs (Leitinger and Kwan, 2006; Dengjel et al., 2007). Both receptors are commonly activated by various types of collagen (mainly type I) but distinctly activated by type IV for DDR1 and type X for DDR2 (Dengjel et al., 2007; Avraham and Yarden, 2011). Surprisingly, the substitution of five peripheral amino acids in DDR2 with their DDR1 counterparts converts DDR2 into a receptor of type IV collagen (Xu et al., 2011). Another intriguing feature of DDRs is their unusually slow autophosphorylation upon stimulation by the ligand compared with typical RTKs (hours rather than seconds; Dengjel et al., 2007). Furthermore, DDRs dimerization is essential for collagen recognition unlike other RTKs in which ligand binding leads to receptor dimerization (Leitinger, 2003). By contrast to DDR2 which binds to several collagen peptides (Farndale et al., 2008), DDR1 binding is restricted to the GVMGFO motif (Gu et al., 2011). This collagen binding site of DDRs is highly conserved, 11 of the 13 amino acids identified by nuclear magnetic resonance (NMR) by Ichikawa and co-workers are identical between DDR1 and DDR2 (Ichikawa et al., 2007).

Upon collagen binding, specific tyrosines residues present in the activation loop of DDRs tyrosine kinase domain are phosphorylated. Phosphorylation of these tyrosine residues leads to the binding of a number of different Src homology 2 (SH2) and phosphotyrosine binding (PTB) domain containing proteins (Carafoli and Hohenester, 2013). Moreover, the activated KD of DDRs is believed to autophosphorylate several tyrosines in the JM region, which serve as docking sites for several adaptor proteins such as SH2 domain containing transforming protein 1 (Shc1) (Ikeda et al., 2002), cytoplasmic protein Nck2 (Koo et al., 2006), protein tyrosine phosphatase SHP-2 (Wang et al., 2006), cell division control protein 42 (Cdc42) (Yeh et al., 2009), nuclear factor kappa-light-chain-enhancer of activated B cells (NF-κB) (Das et al., 2006), extracellular signal-regulated kinase mitogen-activated protein kinase (ERK1/2-MAPK) (Su et al., 2009), activator protein (AP)-1 (Su et al., 2009), and members of the signal transducers and activators of transcription (STAT) family of transcription factors (Wang et al., 2006). Defining DDR signaling pathways has always been a challenging task. Indeed, DDRs bind to multiple collagens and both exhibit unique and common structural and activation properties, but phosphorylate different target receptors (Ongusaha et al., 2003). In addition, DDRs may act in concert with other signaling receptors, including the Wnt5a/Frizzled (Dejmek et al., 2003) and Notch1 (Kim et al., 2011) receptors in the case of DDR1 and the insulin receptor (Iwai et al., 2013a) in the case of DDR2. Finally, DDRs signaling is cell/tissue type-specific and context-dependent. For example, DDR1 inhibits cell migration in Madin-Darby canine kidney (MDCK) cells (Wang et al., 2006) whereas, in other cellular systems, DDR1 and DDR2 promote cell migration and/or invasion (Yoshida and Teramoto, 2007).

## DDRs implication in cancer (Tables 1, 2)

**Figure 2:**
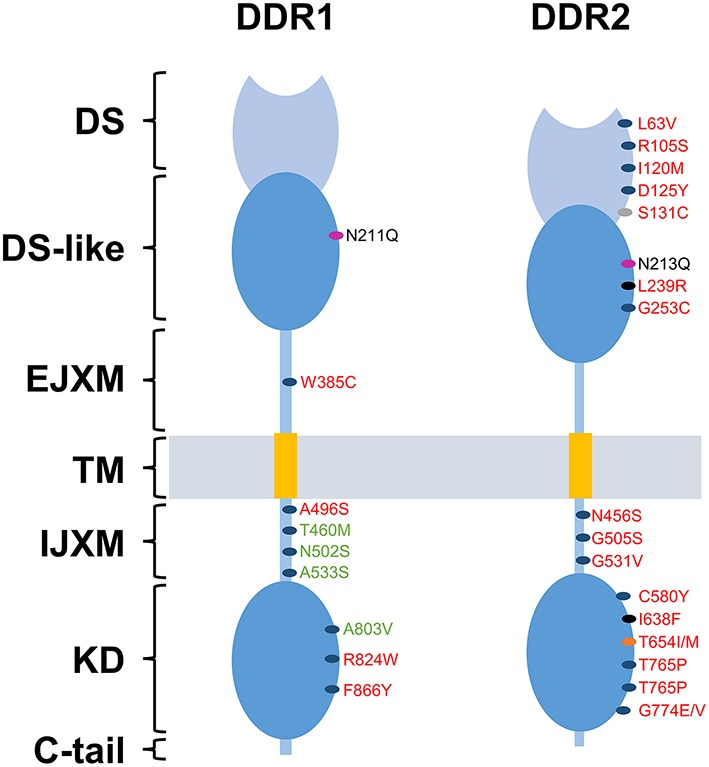
**DDRs reported mutations in cancer**. Domain distribution of somatic mutations of mutated DDR1 and DDR2 identified in lung cancer (red) and Acute Myeloid Leukemia (green) samples. Blue spots represent mutation with undefined role. Gray spot represent a mutation that promotes tumor cell proliferation and invasion. Pink spots represent a mutation that activates constitutively DDRs. Black spots represent mutations responsible for an increased sensitivity to dasatinib. Orange spot represents the “gatekeeper mutation,” responsible for the resistance of tumor cells to dasatinib. DS, discoidin domain; DS-like, discoidin-like domain; EJXM, extracellular juxtamembrane region; TM, transmembrane segment; IJXM, intracellular juxtamembrane region; KD, kinase domain.

DDRs have been linked to tumor progression in several human cancers. In fact, several studies have shown that the expression and activation of DDRs are often dysregulated in such diseases (Valiathan et al., 2012). In addition, somatic mutations of DDRs genes have been found in various cancers (Ford et al., 2007). In the case of DDR2, these mutations are present in 3–4% of patients with lung squamous cell cancer (Hammerman et al., 2011) and have been reported in other cancers at comparable frequencies including lung adenocarcinoma, cervical carcinoma, gastric carcinoma, bladder carcinoma, melanoma, colorectal cancer, head, and neck cancer (Beauchamp et al., 2014). Mutation known as I638F, has been shown to promote resistance to inhibitors of DDR2 kinase function (Hammerman et al., 2011; Figure 2). Nevertheless, the picture is still complicated by the fact that DDRs can act also as anti-tumorigenic receptors and their effect is highly dependent on the type of cancer and the nature of the microenvironment. In the following parts, we will try to explore recent data on the role of DDR1 and DDR2 in malignant transformation, cell proliferation, epithelial to mesenchymal transition, migratory, and invasive processes.

**Table 1:** **Non-exhaustive list of reported DDRs *in vitro* functions in various aspects of cancer progression**.

		**DDR1 in cancer cells**	**DDR2 in cancer cells**
Positive regulator	Proliferation /survival	- Human glioma: U251, GI-1 and T98G (Yamanaka et al., 2006) - Human pancreatic adenocarcinoma: BXPC3 (Rudra-Ganguly et al., 2014) - Human colon carcinoma: HCT116 (Ongusaha et al., 2003; Kim et al., 2011) - Human osteosarcoma: Saos2 (Ongusaha et al., 2003) - Human breast cancer: MCF-7 (Ongusaha et al., 2003), MDA-MB-435 and T47D (Das et al., 2006) - Human Hodgkin lymphoma: L428 (Cader et al., 2013).	- Human squamous cell lung cancer: H2286 and HCC366 (Hammerman et al., 2011) - Human osteosarcoma: U2OS (Han et al., 2014) - Human squamous cell lung cancer: H1299 (Kim et al., 2014) - Human melanoma: A375 (Badiola et al., 2011) - Human hepatoma: SKHEP (Badiola et al., 2011) - Human colon carcinoma: HT-29 (Badiola et al., 2011).
	EMT	- Human hepatoma: HAK-1A and HAK-1B (Maeyama et al., 2008) - Human non-small cell lung carcinoma: A549 (Walsh et al., 2011) - Human colorectal cancer: LOVE1 and LOVO (Hu et al., 2014) - Human pancreatic adenocarcinoma: BxPC3 (Shintani et al., 2008).	- Human lung carcinoma: A549 (Walsh et al., 2011) - Human breast cancer: MDA-MB-231 (Zhang et al., 2013; Ren et al., 2014), MCF-7 and MDA-MB-468 (Ren et al., 2014).
	Migration	- Human glioma: G140 (Ram et al., 2006) - Human hepatocellular carcinoma: HLE and Huh-7 (Park et al., 2007) - Human non-small cell lung carcinoma: A549 and H358 (Yang et al., 2010) - Human pancreatic cancer: BxPC3 (Rudra-Ganguly et al., 2014) - Human colorectal cancer: LOVE1 and LOVO (Hu et al., 2014) - Human breast cancer: MCF-7 (Huang et al., 2009), MDA-MB-231 (Castro-Sanchez et al., 2010), MDA-MB-468 and T47D (Neuhaus et al., 2011).	- Human melanoma: A375 (Badiola et al., 2011) - Human hepatoma: SKHEP (Badiola et al., 2011) - Human colon carcinoma: HT-29 (Badiola et al., 2011) - Human prostate cancer: PC-3 (Yan et al., 2014) - Human lung carcinoma: A549 (Walsh et al., 2011) - Human nasopharyngeal carcinoma isolated cells (Chua et al., 2008) - Murine melanoma: B16BL6 (Poudel et al., 2015).
	Invasion	- Human glioma: G140 (Ram et al., 2006) - Human hepatocellular carcinoma: HLE and Huh-7 (Park et al., 2007) - Human oral squamous cell carcinoma: A431 (Hidalgo-Carcedo et al., 2011) - Human colorectal cancer: LOVE1 and LOVO (Hu et al., 2014) - Human non-small cell lung carcinoma: A549 (Yang et al., 2010; Miao et al., 2013; Juin et al., 2014) and H358 (Yang et al., 2010) - Human hepatoblastoma: Huh6 (Juin et al., 2014) - Human breast cancer: MDA-MB-231 (Castro-Sanchez et al., 2011; Juin et al., 2014) - Human prostate cancer: PC-3 (Shimada et al., 2008) - Human pituitary adenoma: HP-75 (Yoshida and Teramoto, 2007).	- Human prostate cancer: LNCaP and PC-3 (Yan et al., 2014) - Human squamous cell lung cancer: H1299 (Kim et al., 2014) - Human breast cancer: MDA-MB-231 (Zhang et al., 2013) - Murine melanoma: B16BL6 (Poudel et al., 2015).
Negative regulator	Proliferation /survival	- Human breast cancer: MCF-7 and ZR-75-1 (Maquoi et al., 2012; Assent et al., 2015).	- Human melanoma: A2058 (Wall et al., 2005) and M24met (Henriet et al., 2000; Wall et al., 2005, 2007) - Human fibrosarcoma: HT-1080 (Wall et al., 2005) - Human squamous cell lung cancer: (Iwai et al., 2013b; Miao et al., 2014).
	EMT	- Human breast cancer: Hs578T, MCF-7 and MDA-MB-231 (Koh et al., 2015).	NR
	Migration	- Human breast cancer: MCF-7 (Hansen et al., 2006), MDA-MB-231 (Hansen et al., 2006; Koh et al., 2015) and Hs578T (Koh et al., 2015).	- Murine colon carcinoma: MCA38 (Badiola et al., 2012).
	Invasion	NR	NR

*DDR, Discoidin domain receptor; EMT, Epithelial mesenchymal transition; NR, not reported*.

**Table 2:** **Insights into DDRs contribution in cancer from *in vivo* studies**.

	**Cancer cell type**	***In-vivo*** **model**	**Reported Results**	**References**
DDR1	Human pancreatic adenocarcinoma	BXPC3 mouse tumor xenografts	shRNA-DDR1 silencing reduced the growth of tumor xenografts (~50% reduction compared to control)	Rudra-Ganguly et al., 2014
	Human colon carcinoma	HCT116 mouse tumor xenografts	shRNA-DDR1 silencing reduced the growth of tumor xenografts (~30% reduction compared to control)	Kim et al., 2011
	Human breast cancer	Hs587T and MDA-MB-231 cells seeded on upper layer of Chorioallantoic membrane (CAM)	DDR1 overexpression in cells, induced a decreased invasion after 48 h of incubation	Koh et al., 2015
	Human prostate cancer	Androgen independent-LNCaP and LNCaP prostate seeded on CAM	siRNA-DDR1 silencing in cells, induced a decreased invasion after 72 h of incubation	Shimada et al., 2008
DDR2	Human squamous cell lung cancer (SCC)	NCI-H1703, NCI-H2286 or A549 cells athymic nude mouse xenografts	Dasatinib inhibited the proliferation of DDR2-mutated SCC cell lines (NCI-H1703, NCI-H2286 but not A549) in xenograft studies	Hammerman et al., 2011
	Human melanoma	Intrasplenic inoculation of A375R2-70 and A37R2-40 cells in C57BL/6J-Hfn11 nude mice	siRNA-DDR2 silencing in A375R2-70 and -40 reduced experimental liver metastasis development, by 60 and 75%, respectively	Badiola et al., 2011
	Mouse breast cancer	4T1-Luc/GFP cells implantation into the breast tissue of syngeneic Balb/cJ mice	DDR2 depletion led to a reduced metastatic capacity of 4T1-Luc cells	Zhang et al., 2013
	Human breast cancer	MDA-MB-231-luc-D3H2LN cells transplantation into nude mice mammary fat pads	shRNA-DDR2 silencing improved mice lifespan and attenuated cells invasive capacity even 7 weeks after transplantation	Ren et al., 2014
	Human prostate cancer	PC-3 cells intrabone injection in mice metastasis model	DDR2 depletion alleviated PC-3 cells induced osteolytic lesions, signature of bone destruction	Yan et al., 2014
	Murine colon carcinoma	Intrasplenic MCA38 cells injection into DDR2-deficient mice	Increase in cancer cells hepatic colonization efficiency (hepatic occupied volume and number of metastatic foci per area unit)	Badiola et al., 2012

*shRNA, Short hairpin ribonucleic acid; siRNA, small interfering ribonucleic acid*.

## Cell proliferation and survival

The emerging role of DDRs in tumor *cell survival/proliferation* and their crosstalk with oncogenic signaling was previously evaluated through *in vitro* and/or *in vivo* DDRs silencing strategies. Both DDR1 and DDR2 can exhibit pro- (Ongusaha et al., 2003; Das et al., 2006; Yamanaka et al., 2006; Hammerman et al., 2011; Kim et al., 2011, 2014; Cader et al., 2013; Han et al., 2014; Rudra-Ganguly et al., 2014) and anti- (Wall et al., 2005, 2007; Assent et al., 2015) proliferative activities in a cell type and context-dependent manner. Targeting DDR1 with siRNA in glioma (Yamanaka et al., 2006) and pancreatic adenocarcinoma cell lines (Rudra-Ganguly et al., 2014) inhibited tumor cell proliferation *in vitro* and impaired subcutaneous xenograft tumor growth in mice. Upregulation of transforming growth factor beta 1 (TGFβ1), following DDR1 silencing, is thought to induce tumor cell growth arrest (Rudra-Ganguly et al., 2014). Furthermore, inhibition of DDR1 in human colon carcinoma cells (Ongusaha et al., 2003), breast cancer cell lines (Ongusaha et al., 2003; Das et al., 2006) and collagen treated Hodgkin lymphoma cells (Cader et al., 2013) resulted in an increase in cell death in response to induced DNA damage (Ongusaha et al., 2003; Das et al., 2006; Cader et al., 2013). These data suggest that DDR1 expression in tumor cells can confer resistance to chemotherapeutic drugs. This resistance is thought to be due to an activation of NFκB and its downstream effectors, including cyclooxygenase-2 (COX-2) which plays a role in resistance to chemotherapy-induced apoptosis (Cao and Prescott, 2002; Das et al., 2006) or by counteracting p53 mediated apoptosis (Ongusaha et al., 2003). Consistently, studies in human HCT116 colon carcinoma cells showed that DDR1, in response to collagen-induced activation, promotes cell survival in a Notch signaling manner (Kim et al., 2011).

In the case of DDR2, mutations of the receptor were shown to promote cell growth in NIH3T3 mouse embryonic fibroblast cells [(Hammerman et al., 2011) section Supplementary Data, Figure S3-a]. An upregulation of DDR2 followed by an increase in cell proliferation and survival is thought to be induced by an overexpression of COX-2 in U2OS human osteosarcoma cells (Han et al., 2014). In H1299 cells, inhibition of DDR2 activity by overexpressing the juxtamembrane domain containing JM2 suppressed collagen-induced colony formation and cell proliferation. JM2-mediated DDR2 dimerization is likely to be essential for activation of the receptor and cell proliferation. Thus, inhibition of DDR2 function using a JM2-containing peptide may be a useful strategy for the treatment of DDR2-positive cancers (Kim et al., 2014).

In contrast to the above studies, DDR1 and DDR2 can also act as cell growth inhibitors. Indeed, DDR2 has been reported to induce an inhibitory effect on proliferation of human melanoma and fibrosarcoma cells, once cultured on fibrillar collagen, with a growth arrest in the G0/G1 phase of the cell cycle. This process was shown to be induced through p15^INK4b^ cyclin-dependent kinase inhibitor, raising the question whether p15^INK4b^ could be a downstream target of DDR2 signaling (Henriet et al., 2000; Wall et al., 2005, 2007). SHP-2 has been shown to be a key downstream component of DDR2 signaling. Indeed, Iwaï and co-workers demonstrated that the mutation I638F in the kinase domain of DDR2, leads to an inhibition of SHP-2 phosphorylation and a loss of its cell growth suppression effect, whereas mutations L63V in the discoidin domain and G505S in the intracellular juxtamembrane region don't have any effect on SHP-2 phosphorylation (Iwai et al., 2013b). In addition, the mutation S131C in the DS domain of DDR2 was able to increase squamous cell lung cancer (SCC) proliferation *in vitro* and *in vivo* (Miao et al., 2014; Figure 2). Related to DDR1, it has been identified as a key sensor that monitors the cellular microenvironment and triggers apoptosis through the induction of the pro-apoptotic Bcl-2-interacting killer protein (BIK) in luminal breast cancer cells within a collagen three dimensional culture system (Maquoi et al., 2012; Assent et al., 2015).

## Epithelial/mesenchymal transition

The epithelial to mesenchymal transition (EMT) plays crucial role in the differentiation of multiple tissues and organs. EMT also contributes to tissue repair, but it can adversely cause organ fibrosis and promote tumor progression through a variety of mechanisms. EMT is characterized by an increase in cell motility and invasiveness, induction and maintenance of stem cell properties, prevention of apoptosis, senescence, and resistance to therapy (Thiery et al., 2009). Tumor cells that undergo EMT are found to express less epithelial markers such as E-cadherin (Maeyama et al., 2008; Walsh et al., 2011; Zhang et al., 2013; Hu et al., 2014; Ren et al., 2014; Koh et al., 2015) and cytokeratins (Maeyama et al., 2008) but express more mesenchymal markers such as vimentin (Maeyama et al., 2008; Walsh et al., 2011; Hu et al., 2014; Ren et al., 2014; Koh et al., 2015) and N-cadherin (Shintani et al., 2008; Hu et al., 2014), with a possible switch in DDR expression from DDR1 (epithelial) to DDR2 (mesenchymal). These reports have shown that induction of an EMT phenotype results in transcriptional downregulation of DDR1 and that a predominant DDR2 expression reflects a result of EMT process toward more malignant cells (Maeyama et al., 2008; Toy et al., 2015). In addition, the newly expressed DDR2, in several cell lines of human cancer such as liver (HAK-1A and HAK-1B cells) (Maeyama et al., 2008), lung (A549 cells) (Walsh et al., 2011), and breast (MDA-MB-231, MCF-7, SK-BR3, and MDA-MB-468 cells) (Zhang et al., 2013; Ren et al., 2014), is phosphorylated upon interaction with type I collagen, suggesting that the induced receptor is physiologically active.

Studies in A549 lung carcinoma cells showed that inhibiting the expression of DDR2 with siRNA is sufficient to alter activity of the NF-κB and the lymphoid enhancer-binding factor 1 (LEF-1) transcription factors and to inhibit EMT and cell migration induced by TGF-β1 (Walsh et al., 2011). While in breast cancer cells, Zhang and co-workers showed that activation of DDR2 regulates SNAIL1 protein stability by stimulating ERK2 activity, in a Src-dependent manner. Activated ERK2 directly phosphorylates SNAIL1, leading to SNAIL1 nuclear accumulation, a decrease in ubiquitination, and an increase in protein half-life. Thus, DDR2 maintains SNAIL1 protein level and its activity in tumor cells, facilitating cell invasion (Zhang et al., 2013). Lately, it has been showed that DDR2 expression and activation in breast cancer cells can be increased by hypoxia, which is well-known to participate in tumor metastatic events (Ren et al., 2014).

While these studies suggest that acquisition of a more mesenchymal-like phenotype is associated with expression of DDR2, other studies suggest that, depending on the cell type, both DDRs can promote EMT. DDR1 has been shown to interact with α2β1 integrin receptors and activate cell signaling pathways, which promote expression of mesenchymal markers (Shintani et al., 2008). Many studies have shown that microRNAs can also regulate the expression of various genes closely associated with invasion and metastasis in colorectal cancer (CRC) pathogenesis. Indeed, overexpressing miR-199a-5p leads to a decrease in the expression of DDR1, matrix metalloproteinase-2 (MMP-2), N-cadherin, and vimentin, and an increase in E-cadherin expression in both LOVE1 and LOVO CRC cell lines. However, down-regulation of miR-199a-5p resulted in the opposite effects (Hu et al., 2014). Zinc finger E-box-binding homeobox 1 (ZEB1) is a transcription factor that is overexpressed downstream of EMT inducers, and plays a critical role in mediating changes in gene expression during EMT, particularly for E-cadherin (Eger et al., 2005). Studies in triple-negative breast cancer cells revealed a novel H-Ras/ZEB1/DDR1 network that contributes to breast cancer progression in Ras-dependent hyperactive signaling. These data showed that oncogenic H-Ras signaling upregulates ZEB1, which in turn suppresses E-cadherin and DDR1, leading to EMT and invasion (Koh et al., 2015).

## Cell migration

As mentioned above, both DDR1 and DDR2 can support EMT and contribute to the adaptation of cells to their new environment by activation, in addition to other receptors, of EMT-induced migration programs. Regulation of cell migration by DDR1 was reported in various cancer cell lines including glioma (Ram et al., 2006), hepatocarcinoma (Park et al., 2007), lung (Yang et al., 2010), pancreas (Rudra-Ganguly et al., 2014), colorectal (Hu et al., 2014), and breast (Hansen et al., 2006; Huang et al., 2009; Castro-Sanchez et al., 2010; Neuhaus et al., 2011) carcinoma. Nevertheless, this regulation is cell type and receptor isoform dependent. Therefore, conflicting reports attributed inhibitory (Hansen et al., 2006; Koh et al., 2015) as well as pro-migratory (Ram et al., 2006; Park et al., 2007; Huang et al., 2009; Castro-Sanchez et al., 2010; Yang et al., 2010; Neuhaus et al., 2011; Hu et al., 2014; Rudra-Ganguly et al., 2014) effects for DDR1 in cell migration. Overexpression of DDR1a (but not DDR1b) in glioma (Ram et al., 2006), hepatocellular carcinoma (Park et al., 2007) and non-small lung cancer cells (Yang et al., 2010) significantly promotes tumor cell motility. Although, the significance of the difference between the migration induced effects of DDR1a and DDR1b is unknown, structural differences and divergent signaling between DDR1a and DDR1b have been suggested (Park et al., 2007). As an essential soluble component of the ECM, TGF-β1 elicits numerous changes in cellular behavior but has a conflicting role in cancer progression. Studies on pancreatic cancer cells showed that the pro-migratory effect of DDR1, in these cells, appears to be in part mediated *via* TGF-β1 downregulation (Rudra-Ganguly et al., 2014). Stimulation of MDA-MB-231 breast cancer cells with type IV collagen is able to induce cell migration through a DDR1 and CD9-dependent pathway (Castro-Sanchez et al., 2010). In MDA-MB-468 breast cancer cells, DDR1-dependent promotion of cell migration was shown to be induced through a regulation of the migration suppressor tyrosine-protein kinase (SYK) activity (Neuhaus et al., 2011). In MCF-7 cells, full-length DDR1 associated to myosin IIA facilitates the process of cell migration (Huang et al., 2009). DDR1 can also play a negative role in cell migration. Indeed, overexpression of DDR1 in Hs587T breast cancer cells reduced their *in vitro* migratory behavior in type I collagen three dimensional (3D) culture system (Koh et al., 2015). While in MDA-MB-231 breast cancer cells, DDR1 suppresses migration only when co-expressed with its interacting partners, the Dopamine and cAMP-regulated neuronal phosphoprotein-32 (DARPP-32) (Hansen et al., 2006).

DDR2, when activated by type I collagen, was shown to support the migration of human A375 and B16BL6 murine melanoma cells (Badiola et al., 2011; Poudel et al., 2015), SK-HEP hepatoma cells, HT-29 colon carcinoma cells (Badiola et al., 2011), PC-3 prostate cancer cells (Yan et al., 2014), A549 lung carcinoma cells (Walsh et al., 2011), and nasopharyngeal carcinoma cells (Chua et al., 2008). Badiola and co-workers showed, that the c-Jun N-terminal kinases (JNK) pathway is involved in DDR2 inducing cell migration in A375 human melanoma cells (Badiola et al., 2011). While in A549 lung cancer cells, DDR2 inhibition with siRNA was sufficient to inhibit cell migration induced by TGF-β1 (Walsh et al., 2011). Recently, it has been shown that DDR2 promotes migratory phenotype of B16BL6 murine melanoma cells through the regulation of ERK and NF-κB signaling pathways (Poudel et al., 2015). In a single report, DDR2 was shown to be a negative migration regulator. Indeed, culturing MCA38 colon carcinoma cells in presence of conditioned media from untreated DDR2^−∕−^ hepatic stellate cells (HSCs) and tumor-activated DDR2^−∕−^ HSCs was able to enhance the migration of MCA38 cells, respectively, by 60 and 90% (Badiola et al., 2012).

## Cell invasion

Tumor invasion is a complex process that requires ECM degradation and tissue remodeling. Indeed, this process requires the activation of multiple genes but depends also on the action of key molecules such as ECM-degrading proteases and ECM receptors. Among these receptors, DDR1 has been found to be highly expressed in invasive tumors indicating its critical role as a regulator of cell invasion and subsequent tumor metastasis (Valiathan et al., 2012). Moreover, accumulating evidence using Matrigel or type I collagen as matrix barriers suggests that DDR1 plays a promoting role in invasion of a variety of human cancers including glioma (Ram et al., 2006), hepatocellular (Park et al., 2007), squamous epidermoid (Hidalgo-Carcedo et al., 2011), colorectal (Hu et al., 2014), lung (Yang et al., 2010; Miao et al., 2013; Juin et al., 2014), prostate (Shimada et al., 2008), breast carcinomas (Castro-Sanchez et al., 2011; Juin et al., 2014). This has also been observed for pituitary adenoma (Yoshida and Teramoto, 2007). Matrix metalloproteinases (MMPs), a family of zinc-dependent endopeptidases, degrade the basement membrane and ECM, facilitating cell migration, tumor invasion, and metastasis. Among MMPs, MMP-2, and MMP-9 are considered important in the malignant behavior of tumor cells (Shuman Moss et al., 2012). Several reports showed that DDR1 can function as an inducer of MMP-2 (Ram et al., 2006; Park et al., 2007; Yoshida and Teramoto, 2007; Castro-Sanchez et al., 2011; Hu et al., 2014; Juin et al., 2014), or/and MMP-9 (Park et al., 2007; Yoshida and Teramoto, 2007; Shimada et al., 2008; Yang et al., 2010; Castro-Sanchez et al., 2011; Miao et al., 2013) and thus, contribute to the matrix components degradation. Overexpression of the DDR1a but not DDR1b isoform confers an aggressive invasive behavior to glioma cells *in vitro* by increasing their ability to invade Matrigel or type I collagen. DDR1a activation by collagen leads to the conversion of pro-MMP-2 (72 kDa) into its active form (62 kDa) (Ram et al., 2006). Whereas, DDR1a and DDR1b overexpression resulted in an increase of MMP-2 and MMP-9 in hepatocellular carcinoma and non-small lung cancer cell lines, respectively (Park et al., 2007; Yang et al., 2010). Hu and co-workers showed that overexpression of DDR1 induces invasion in colon carcinoma through the up-regulation of MMP-2 (Hu et al., 2014). By contrast, DDR1 in pituitary adenoma cell line induced an increase in both MMP-2 and MMP-9 secretion (Yoshida and Teramoto, 2007). In 2011, Hidalgo-Carcedo and co-workers suggested that the ability of DDR1 to support collective cell invasion of human A431 oral squamous cell carcinoma cells does not require receptor signaling and is independent of its activation by collagen. In these cells, DDR1 through its interaction with the cell polarity regulators Par3 and Par6, induces a decrease in actomyosin contractility and thereby enables collective cancer cell invasion (Hidalgo-Carcedo et al., 2011). Prostate cancer antigen-1 (PCA-1) has been shown to contribute to prostate carcinoma cell invasion through DDR1 (Shimada et al., 2008). In MDA-MB-231 breast cancer cells, type IV collagen induces MMP-2 and MMP-9 secretion and invasion through a DDR1 and Src-dependent pathway (Castro-Sanchez et al., 2011). Moreover, MMP-2 and MMP-9 secretion required protein kinase C (PKC) activity and epidermal growth factor receptor (EGFR) activation (Castro-Sanchez et al., 2011). Frederic Saltel's team proposed that DDR1 could be a sensor used by MDA-MB-231 breast and A549 lung carcinoma cells to interact with fibrillar type I collagen, leading to the organization of linear invadosomes, *via* a Cdc42-Tuba pathway (Juin et al., 2014). Neither DDR1 kinase activity nor Src tyrosine kinase were required for the formation and activity of invadosomes (Juin et al., 2014).

DDR2 has been found to promote invasion in prostate (Yan et al., 2014), non-small cell lung (Kim et al., 2014), breast (Zhang et al., 2013), and metastatic melanoma (Poudel et al., 2015). Zhang and co-workers have identified an intracellular signaling pathway initiated by collagen-mediated DDR2 activation, leading to ERK1/2 activation in a Src-dependent manner and SNAIL1 phosphorylation. This induced SNAIL1 stabilization and promoted MDA-MB-231 cell invasion *in vitro* and *in vivo* (Zhang et al., 2013). Recently, Poudel and co-workers demonstrated that DDR2 was able to modulate MMP-2 and MMP-9 secretion in response to type I collagen and to regulate the invasive phenotype of murine metastatic melanoma cells through a regulation of ERK1/2 and NF-κB signaling pathways (Poudel et al., 2015).

## DDRs inhibition and targeted therapy in cancer (Table 3)

The contribution of DDRs in tumor progression clearly indicates that inhibition of these receptors might represent a promising therapeutic strategy. Yet, DDRs inhibitors reported so far are adenosine triphosphate (ATP) competitive inhibitors that bind to either active (type-1 inhibitors) or inactive (type-2 inhibitors) conformations, preventing transfer of the terminal phosphate group of ATP to the protein substrate. Type-1 inhibitors bind in the so-called “open conformation” of DDRs kinase domain, which is characterized by a “DFG-in” configuration of the conserved triad DFG at the beginning of activation loop. In contrast, type-2 inhibitors bind to and stabilize an inactive kinase form that is characterized by “DFG-out” conformation. The “DFG-out” motif opens an additional cavity, a hydrophobic allosteric site that, in addition to the ATP binding pocket, is targeted by type-2 inhibitors (Kothiwale et al., 2015). Using chemical and proteomic approaches, dasatinib, imatinib, nilotinib (Bantscheff et al., 2007; Rix et al., 2007; Day et al., 2008), and ponatinib (Canning et al., 2014) were identified as potent small-molecule inhibitors against DDR1 and DDR2, with IC_50_ values of 0.5, 337, 43, 9 nM and 1.4, 675, 55, 9 nM, respectively (Day et al., 2008; Canning et al., 2014). These four molecules were originally developed to inhibit Bcr-Abl tyrosine kinase in chronic myeloid leukemia. Imatinib, nilotinib, and ponatinib (type-2 inhibitors) are more selective by inhibiting a few tyrosine kinases, whereas dasatinib (type-1 inhibitor) is known to inhibit dozen of tyrosine kinases. In 2011, Hammerman and co-workers have shown that DDR2 is mutated in approximately 4% of lung squamous cell cancer and have reported data to suggest that these mutations induce a gain in DDRs function (Hammerman et al., 2011). The same group has also shown that cell lines harboring these mutations are sensitized to the multitargeted kinase inhibitor dasatinib (Hammerman et al., 2011; Bai et al., 2014). Indeed, dasatinib can efficiently inhibit the proliferation of DDR2- mutated SCC cell lines *in vitro* and *in vivo*, as well as cells ectopically expressing mutant DDR2 (Hammerman et al., 2011). This led to the design of clinical trials testing its efficacy in patients with non-small-cell lung carcinoma (NSCLC) (Haura et al., 2010; Johnson et al., 2010; Pitini et al., 2013; Gold et al., 2014). However, inhibition of DDRs signaling pathways often activates secondary survival mechanisms (Beauchamp et al., 2014). Therefore, combining dasatinib and Src kinase inhibitors, could enhance the efficacy of dasatinib in NSCLC (Khurshid et al., 2012) and could also decrease substantial toxicity associated with dasatinib when administered alone (Brunner et al., 2013; Dy and Adjei, 2013).

**Table 3:** **An update on DDRs inhibitors: compound name, type and reported half maximal inhibitory concentration**.

**Compound**	**Half maximal inhibitory concentration (IC**_50_**) nM**		**Inhibitor type**
	**DDR1**	**DDR2**	
Dasatinib (Day et al., 2008)	0.5 ± 0.2 nM	1.4 ± 0.3 nM	Kinase type I inhibitor
Nilotinib (Day et al., 2008)	43 ± 3 nM	55 ± 9 nM	Kinase type II inhibitor
Imatinib (Day et al., 2008)	337 ± 56 nM	675 ± 127 nM	Kinase type II inhibitor
Ponatinib (Canning et al., 2014)	9 nM	9 nM	Kinase type II inhibitor
Actinomycin D (Siddiqui et al., 2009)	NR	9000 nM	Antibiotic
LCB 03-0110 (Sun et al., 2012)	164 nM	171 nM	Thienopyridine derivative
7rh (Gao et al., 2013)	6.8 nM	101.4 nM	3-(2-(pyrazolo[1,5-a]pyrimidin-6-yl)-ethynyl)benzamides derivative
7rj (Gao et al., 2013)	7 nM	93.6 nM	3-(2-(pyrazolo[1,5-a]pyrimidin-6-yl)-ethynyl)benzamides derivative
2a (Richters et al., 2014)	68 nM	65 nM	Pyrazolo-urea containing compound
4a (Richters et al., 2014)	235 nM	75 nM	Pyrazolo-urea containing compound
4b (Richters et al., 2014)	39 nM	18 nM	Pyrazolo-urea containing compound
DDR1-IN-1 (Kim et al., 2013)	105 nM	413 nM	Kinase type II inhibitor
DDR1-IN-2 (Kim et al., 2013)	47 nM	145 nM	Kinase type II inhibitor

Actinomycin D is an antibiotic compound that has been clinically used for a long time as an anticancer drug to treat rhabdomyosarcoma, Ewing's sarcoma, trophoblastic neoplasia, and testicular carcinoma. Yang and co-workers have identified Actinomycin D as an antagonist of the DDR2-collagen interaction. Indeed, this compound selectively inhibited the activation of DDR2 by type I collagen in HEK293 cells. However, its relatively weak inhibitory activity (IC_50_ = 9000 nM) may limit its further application for inhibition of DDR2 (Siddiqui et al., 2009). LCB 03-0110, a thienopyridine derivative, was identified from a chemical library using the kinase domain of DDR2 and has been shown to inhibit collagen-induced activation of DDR1 and DDR2 receptors with IC_50_ values of 164 and 171 nM, respectively. However, this compound is also an effective inhibitor for other tyrosine kinases (Sun et al., 2012). Moreover, Ding and co-workers have reported a series of 3-(2-(pyrazolo[1,5-a]pyrimidin-6-yl)-ethynyl)benzamides which selectively bind and inhibit, with a type II mode, the kinase function of DDR1 and were significantly less potent against many other kinases such as Bcr-Abl. The two most promising compounds in this series 7rh and 7rj inhibited the kinase activity of DDR1, with IC_50_ values of 6.8 and 7.0 nM, respectively. *In vitro* investigations revealed that these compounds potently inhibited the proliferation of cancer cell lines expressing high levels of DDR1, including A549 and NCI-H23 lung carcinoma, MDA-MB-435, MCF-7, and T47D breast carcinoma and HCT116 colon carcinoma cells (Gao et al., 2013). Using a new strategy called “fluorescent labels in kinases” (FLiK), Rauh and co-workers reached to identify a series of pyrazolo-urea containing compounds as new type II inhibitors of DDR2. The inhibitory effects of three compounds (2a, 4a, and 4b) were further validated by an orthogonal activity-based assay. DDR2 was found to be inhibited, by these compounds, with IC_50_ values of 65, 75, and 18 nM, respectively. These molecules were also able to inhibit DDR1 with IC_50_ values of 68, 235, and 39 nM, respectively. Furthermore, compounds 2a and 4b exhibited significant effect against the T654M gatekeeper mutant of DDR2 with IC_50_ values of 2.0 and 1.0 nM, respectively (Richters et al., 2014). Gray and co-workers designed and synthesized a series of type II inhibitors, among which DDR1-IN-1 and DDR1-IN-2 induced a significant inhibitory effect against DDR1 with IC_50_ values of 105 and 47 nM, respectively. These two inhibitors were also able to inhibit DDR1 activation in U2OS cells in the presence of collagen, with EC_50_ values of 86 and 9.0 nM, respectively (Kim et al., 2013). Using fragment based drug design (so-called back-to-front design), Murray and co-workers have recently discovered novel inhibitors of DDR1 and DDR2 that were potent and selective and displayed interesting pharmacokinetic properties. *In vitro* studies showed that DDR2 activity was highly inhibited by these molecules but in contrast to unselective inhibitors such as dasatinib, they were not able to inhibit proliferation of lung SCC cells harboring a mutant DDR2 (Murray et al., 2015). Finally, other ways to inhibit DDRs consist in the use of targeted delivery of miRNAs based therapeutics such as miR-199a-5p (Hu et al., 2014) or monoclonal antibodies including Fab 3E3 (Carafoli et al., 2012), 48B3 (Ram et al., 2006), and H-126 (Castro-Sanchez et al., 2010) that have been shown to bind to the DS-like domain of DDR1.

## Conclusion

Type I collagen, one of the abundant matrix components and an activator of these receptors, was considered for a long time as a mechanic barrier against cell proliferation and migration but also as a physical obstacle against chemotherapy by decreasing passive diffusion of anticancer drugs. Herein, the reported studies clearly demonstrate that the interaction between type I collagen and DDRs plays a functional role in the regulation of tumor progression, from cell proliferation/survival to migration/invasion processes. However, effects of DDRs activation on tumor progression are controversial. For cell proliferation, it have clearly been demonstrated that DDR1 and DDR2 act as growth suppressors *via* their activation by type I collagen and specific downstream cell signaling. In the case of DDR1, apoptosis was concurrent with cell proliferation suppression. Moreover, the role of DDR2 in the suppression of cell proliferation has been elegantly demonstrated using receptor mutants. In fact, kinase domain mutants were particularly able to alleviate this suppression by inhibiting the activation of these receptors and their downstream cell signaling. These mutations have been identified as novel drivers contributing to cell proliferation *in vivo* and consequently tumor progression. However, other findings strongly suggested that activation of these receptors resulted also in activation of pro-survival signaling pathways. In the case of cell migration and invasion, several *in vitro* and *in vivo* studies specifically addressed the consequences of DDR1 and DDR2 activation in the initiation of migratory and invasive processes. The majority of these studies tended to attribute a functional role of these receptors in the promotion of cell migration and invasion. Moreover, clinical studies on DDR1 and DDR2 expression and the outcome of several cancer pathologies found a correlation between the expression of these receptors, metastasis, and a reduced survival.

Finally, DDR1 and DDR2 are considered as potential therapeutic targets. Therefore, a considerable effort has been made to design inhibitors against these receptors. For kinase activity inhibitors, several molecules including imatinib and nilotinib were identified as inhibitors of these receptors. However, mutations have been noted in several cancer specimens. In the case of DDR2 mutations in squamous lung cell carcinoma, dasatinib showed particular efficacy. Nevertheless, latest *in vitro* model studies have reported a second site mutation in DDR2 which was able to confer resistance to dasatinib. Therefore, and given the clinical trials of dasatinib and other inhibitors in the future, the establishment of additional models of resistance will be important to design strategies that overcome resistance to these molecules.

## Author contributions

All authors listed, have made substantial, direct, and intellectual contribution to the work, and approved it for publication.

## Funding

This work was supported by grants from Ligue Contre le Cancer 2015 (CCIR Grand-Est). CS is recipient of doctoral fellowships from the French Ministry of Higher Education and Research.

### Conflict of interest statement

The authors declare that the research was conducted in the absence of any commercial or financial relationships that could be construed as a potential conflict of interest.
