# Expression of Concern: Discoidin Domain Receptors: Potential Actors and Targets in Cancer

**DOI:** 10.3389/fphar.2016.00228

**Published:** 2016-07-21

**Authors:** Frontiers Editorial Office

With this notice, Frontiers states its awareness of concerns regarding a number of instances of failure to adequately cite or attribute credit appropriately within the above-referenced article. Prof. Theophile Godfraind and Prof. Olivier Feron, Chief Editors of the journal and specialty section respectively, will direct an investigation in full accordance with our procedures. The situation will be updated as soon as the investigation is complete.

