# Corrigendum: Discoidin Domain Receptors: Potential Actors and Targets in Cancer

**DOI:** 10.3389/fphar.2016.00346

**Published:** 2016-09-30

**Authors:** Hassan Rammal, Charles Saby, Kevin Magnien, Laurence Van-Gulick, Roselyne Garnotel, Emilie Buache, Hassan El Btaouri, Pierre Jeannesson, Hamid Morjani

**Affiliations:** Extracellular Matrix and Cellular Dynamics, Faculty of Pharmacy, MEDyC Centre National de la Recherche Scientifique UMR7369, Reims UniversityReims, France

**Keywords:** discoidin domain receptors, tyrosine kinase, extracellular matrix, collagen, cell signaling, cancer, targeted therapy

## Corrigendum

Following relevant reader's comments and Editor's request, my co-authors and I would like to add some references which have been unintentionally omitted in some sections of the review. We thank the readers and the Editorial Office for pointing this out and helping us to improve the manuscript.

## “Introduction section” (page 2)

After surgery, radiation therapy (RT) has long been an integral component of cancer care. It is usually employed to locally eradicate tumor cells as well as alter tumor stroma with either curative or palliative intent (Hodge et al., 2012; Kwilas et al., 2012).

Therefore, current efforts have been focusing on understanding the molecular, cellular, and systemic processes driving cancer initiation, progression, heterogeneity, and metastatic spread (Ramos and Bentires-Alj, 2015; Semenova et al., 2015).

As a major part of the tumor ECM, type I collagen exhibits high density and altered architecture in malignant cancer and is causally linked to tumor formation and metastasis (Ren et al., 2014).

Until recently, these effects on tumor cells were exclusively attributed to integrins, a major class of receptors that mediate cell interactions with extracellular matrix components. The identification of the Discoidin Domain Receptor (DDR) family as collagen receptors represents a new paradigm in the regulation of collagen-cell interactions and regulation of tumor progression (Marquez and Olaso, 2014).

DDR1 and DDR2 were initially discovered by homology cloning based on their catalytic kinase domains and were orphan receptors until 1997 when Shrivastava and co-workers and Vogel and co-workers, reported that different types of collagen are functional ligands for these receptors (Leitinger, 2014).

In contrast with classical growth factor tyrosine kinase receptors such as the epithelial growth factor receptor (EGFR) and fibroblast growth factor receptor (FGFR) which display a rapid and transient activation, DDR1 and DDR2 are unique in that they exhibit remarkably delayed and sustained receptor phosphorylation upon binding to collagen (Iwai et al., 2013).

Furthermore, many classical tyrosine kinase receptors (RTKs) undergo negative regulation such as receptor/ligand internalization and subsequent degradation or dephosphorylation by phosphatases (Fu et al., 2013).

In addition, they are uniquely positioned to function as sensors for ECM and to regulate a wide range of cell functions such as migration, cell proliferation, cytokine secretion, and ECM homeostasis/remodeling (Borza and Pozzi, 2014).

## “Structure, function and regulation of DDRs” section (page 3)

DDRs control important aspects of cell behavior including proliferation, migration, adhesion, and ECM remodeling but are dysregulated in various human diseases. They are both activated by several types of collagen. However, this activation strictly requires collagen to be in its native and triple-helical conformation. Heat-denatured collagen is not recognized by DDRs (Valiathan et al., 2012; Carafoli and Hohenester, 2013).

Surprisingly, the substitution of five peripheral amino acids in DDR2 with their DDR1 counterparts converts DDR2 into a receptor of type IV collagen (Carafoli and Hohenester, 2013).

## Author contributions

All authors contributed to the designing, writing and the validation of the last version of the manuscript.

## Funding

This work was supported by grants from Ligue Contre le Cancer 2015 (CCIR Grand-Est). CS is recipient of PhD fellowships from the French Ministry of Higher Education and Research.

### Conflict of interest statement

The authors declare that the research was conducted in the absence of any commercial or financial relationships that could be construed as a potential conflict of interest.
